# Young Goji Fruit Volatiles Regulate the Oviposition Behavior and Chemosensory Gene Expression of Gravid Female *Neoceratitis asiatica*

**DOI:** 10.3390/ijms252413249

**Published:** 2024-12-10

**Authors:** Hongshuang Wei, Kexin Liu, Jingyi Zhang, Kun Guo, Sai Liu, Changqing Xu, Haili Qiao, Shuqian Tan

**Affiliations:** 1 State Key Laboratory for Quality Ensurance and Sustainable Use of Dao-di Herbs, Institute of Medicinal Plant Development, Chinese Academy of Medical Sciences, Peking Union Medical College, Beijing 100193, China; hswei@implad.ac.cn (H.W.); liukexin202@163.com (K.L.); zhongyaoxili@163.com (J.Z.); kguo@implad.ac.cn (K.G.); sliu@implad.ac.cn (S.L.); cqxu@implad.ac.cn (C.X.); 2 Key Lab of Integrated Pest Management, Department of Entomology, College of Plant Protection, China Agricultural University, Beijing 100193, China

**Keywords:** *Neoceratitis asiatica*, young goji fruit volatiles, oviposition behavior, chemosensory genes, upregulated expression

## Abstract

The goji fruit fly, *Neoceratitis asiatica*, is a major pest on the well-known medicinal plant *Lycium barbarum*. Dissecting the molecular mechanisms of the oviposition selection of *N. asiatica* regarding the host plant will help to identify new strategies for pest fly control. However, the molecular mechanism of chemical communication between the goji fruit fly and the host goji remains unclear. Hence, our study found that young goji fruit volatiles induced the oviposition response of gravid female *N. asiatica*. After *N. asiatica* was exposed to young goji fruit volatiles, the expression of six chemosensory genes (*NasiOBP56h3* and *OBP99a1* in the antennae; *OBP99a2*, *OBP99a3* and *CSP2* in the legs; and *OBP56a* in the ovipositor) was significantly upregulated in different organs of female *N. asiatica* compared with the group without odor treatment according to transcriptome data. Further results of qPCR verification show that the expression levels of the six selected upregulated genes after the flies were exposed to host plant volatiles were mostly consistent with the results of transcriptome data. We concluded that six upregulated genes may be involved in the recognition of young goji fruit volatiles by gravid female *N. asiatica*. Our study preliminarily identifies young goji fruit volatiles as a key factor in the oviposition behavior of *N. asiatica*, which will facilitate further studies on the mechanisms of host oviposition selection in *N. asiatica*.

## 1. Introduction

Goji berry (also named wolfberry and gouqizi) is a traditional Chinese herbal medicine widely loved by consumers at home and abroad [1,2]. Studies have shown that consuming goji berries can reduce age-related vision loss [3], confer resistance to cold temperature [4] and enhance anti-aging properties [5]. Originating from the fact that goji berries are rich in nutrients (mainly including polysaccharides, amino acids and carotene) and medicinal value [3,6], not only do people love to eat them, but so do insects. The goji fruit fly, *Neoceratitis asiatica*, is a major pest during the growth of wolfberry plants, causing up to 80% fruit damage when it infests plants each year [7]. But the control of the goji fruit fly is a difficult problem in the wolfberry industry due to its habit of feeding inside the fruit. Field investigations have found that female goji fruit flies like to lay eggs in young wolfberry fruits. However, the mechanism behind *N. asiatica*’s selection of young wolfberry fruits for laying eggs remains unclear. Therefore, understanding the oviposition selection mechanism between the goji fruit fly and the wolfberry plant will contribute to more effective management of *N. asiatica* in the field.

Insect oviposition behavior plays an important role in the co-evolution of insects and host plants, and maternal oviposition preferences are central to this research [8]. Insect mothers mostly lack the ability to nurture individual offspring and need to choose suitable egg-laying sites and avoid the interference of harmful factors in the environment; therefore, they prefer nutrient-rich egg-laying substrates to ensure the maximum survival rate of their offspring [9]. So how do insect mothers quickly and accurately choose their “delivery rooms”? Some studies have shown that *Drosophila* are capable of integrating taste and tactile information to perceive the hardness and sweetness of egg-laying sites [9,10]. In addition to this, *Drosophila* can sense compounds secreted by its larval body surface to enhance its oviposition site selection preference [11]. Most studies have reported that herbivorous insects such as *Helicoverpa armigera*, *Spodoptera frugiperda* and *Bactrocera dorsalis* seek suitable oviposition sites primarily through the olfactory perception of host plant-specific volatile organic compounds (VOCs) [12,13,14]. For example, the specific component (*Z*)-3-hexenyl-acetate of the corn plant attracts and stimulates *S. frugiperda* to lay their eggs there [13]. Ethyl (*E*, *Z*)-2,4-decadienoate from pear increases the number of eggs deposited by *Cydia pomonella* [15], and *γ*-octalactone from mango can induce female *B. dorsalis* host selection and oviposition stimulation [16,17]. It can be seen that host plant VOCs play a key role in the oviposition selection of herbivorous insects. Research has shown that among the various volatiles identified in *Lycium barbarum*, hexanal and (*E*)-2-hexenal are predominant, accounting for 70–94% of the total composition [18]. Additionally, hexanal, (*E*)-2-hexenal, nonanal, isoamyl alcohol, 1-hexanol, 1-octen-3-ol, ethyl hexanoate, methyl salicylate, ethyl caprylate, o-cymene, d-limonene, linalool, *β*-citral, *β*-elemene and 2-pentylfuran are characteristic aroma volatiles of *L*. *barbarum* [18]. This study focuses on the mechanisms by which these volatiles influence the searching and locating behavior of *N. asiatica*.

Herbivorous insects primarily rely on their antennae to recognize odorant molecules in the air and locate host plants for oviposition [8,19]. The main soluble proteins in the insect olfactory system are odorant-binding proteins (OBPs) and chemosensory proteins (CSPs) [20,21]. OBPs can specifically bind and transport hydrophobic odor molecules corresponding to the receptors, which plays a very important role as the first step in the chemical recognition process of insects [20]. CSPs are similar to OBPs in terms of structure and function and are mainly involved in the recognition and transport of various compounds [8]. Although there is no direct evidence that CSPs are also involved in the chemosensory process of insects, it has been found that CSPs play an important role in the olfactory system of insects [8]. For example, BdorOBP99a is mainly responsible for locating banana host plants, regulating the oviposition selection behavior in *B. dorsalis* [22]. In addition, BdorOBP56d and BdorOBP56d-2 has been shown to be involved in regulating oviposition behavior in *B. dorsalis* [23]. AgosOBP2, responding to the cotton plant VOCs, regulates the oviposition behavior of *Aphis gossypii* females [24]. *Maruca vitrata* MvitOBP3 is strongly bound to host plant-specific components (butyl butyrate, limonene and octyl butyrate) and is considered to be a possible oviposition attractant for female moths on leguminous vegetables [25]. However, the OBPs and CSPs responsible for recognizing host plant volatiles in *N. asiatica* have not yet been identified. Clarifying the recognition mechanisms of these volatile compounds is critical for the development of goji fruit fly behavioral modulators that target olfactory proteins.

Current research on insect egg-laying preferences has focused on agricultural crops [22,23,24,25] and has not yet been reported in medicinal plants. In this study, the oviposition behavior responses of gravid female *N. asiatica* to VOCs (volatile organic compounds) from young goji fruit were detected by using a Y-tube olfactometer and oviposition selection experiments. Then, the transcriptomic data of the antennae, legs and ovipositor of gravid female *N. asiatica* before/after young goji fruit VOC stimulation were obtained by Illumina sequencing. The identification and differential expression of chemosensory genes in different tissue parts of gravid female *N. asiatica* were analyzed by bioinformatics methods. Finally, the expression levels of six upregulated olfactory genes (*NasiOBP56a*, *OBP56h3*, *OBP99a1-a3* and *CSP2*) after VOC exposure were verified by qPCR analysis. The aim of this research is to demonstrate that young goji fruit volatiles can regulate the oviposition selection behavior and olfactory gene expression of gravid female *N. asiatica* for further studies on the molecular mechanism of host oviposition selection in *N. asiatica.*

## 2. Results

### 2.1. Gravid Female N. asiatica Preferred to Lay Eggs on Young Goji Fruit

**Figure 1:**
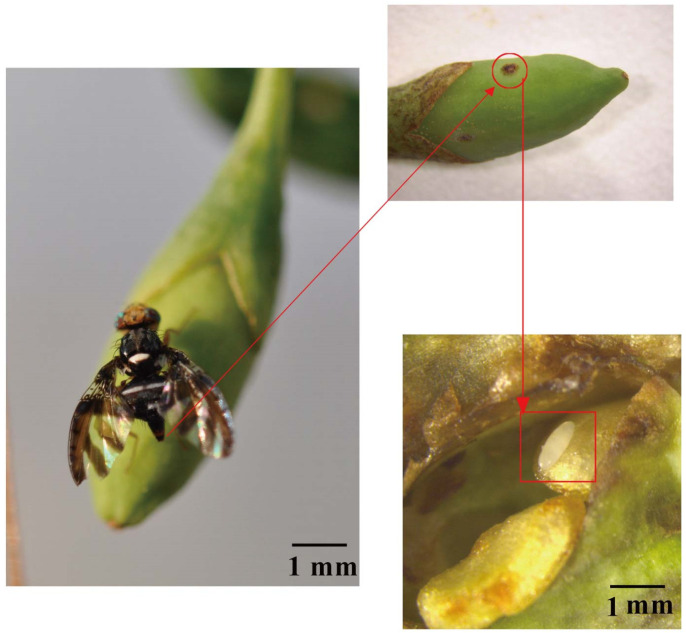
The harmful symptom of female *N. asiatica* laying its eggs on a young goji fruit. The red circle indicates the oviposition site on the goji fruit. The red box indicates the egg of *N. asiatica*.

As shown in Figure 1, a gravid female *N. asiatica* was laying egg on a young goji fruit. After laying eggs, a brown patch formed on the surface of the goji fruit. An egg of *N. asiatica* was hidden beneath this brown patch (Figure 1).

### 2.2. Young Goji Fruit Volatiles Induced Oviposition Behavior Response of Gravid Female N. asiatica

**Figure 2:**
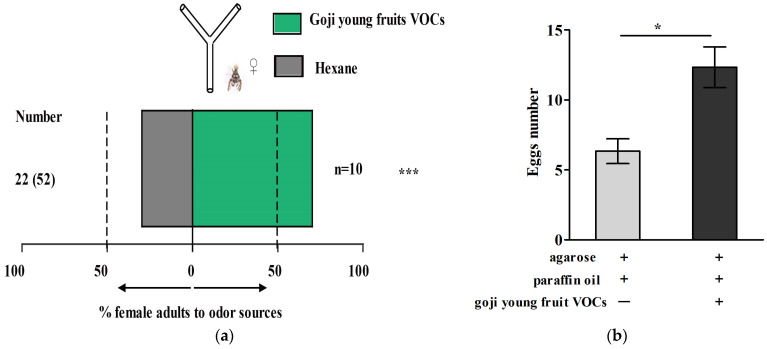
The behavioral assay of the gravid female *N. asiatica*’s response to young goji fruit volatiles. (**a**) The behavioral responses in a Y-tube olfactometer. The mated female flies were tested 4 days after eclosion. The bars represent the overall percentages of female flies choosing either of the odor sources; the number in the bar indicates the total number of female flies choosing the arm. The gray bar represents hexane treatment (control), and the green bar represents fresh young goji fruit volatiles (volatile organic compounds, VOCs) (*** *p* < 0.001, two-sided binominal test). (**b**) The number of eggs laid by the gravid female *N. asiatica* on agarose medium. Treatment groups were placed with a filter paper sheet (1 cm × 1 cm) containing 10 μL of young goji fruit VOCs (the volatiles dissolved into the paraffin oil). Control groups were placed with same-size filter paper containing paraffin oil. The asterisk (*) indicates a significant difference between control and treatment groups (mean ± SE, n = 3, Student’s *t*-test, * *p* < 0.05).

After collecting the young goji fruit volatiles, a behavioral assay of the mated female flies was performed by a Y-tube olfactometer. The results show that the young goji fruit volatiles induced a strong behavioral response in gravid female *N. asiatica* (*p* < 0.001) (Figure 2a). The number of eggs laid by gravid female *N. asiatica* on the agarose medium with young goji fruit VOCs was significantly higher than that on the control groups without fruit VOCs (*p* < 0.05) (Figure 2b).

### 2.3. Transcriptome Analysis of Antennae, Legs and Ovipositor of Gravid Female N. asiatica After Young Goji Fruit Volatile Exposure

#### 2.3.1. Transcriptome Assembly of Gravid Female *N. asiatica* Adults

To further explore the recognition mechanism of *N. asiatica* in response to the volatiles emitted by young goji fruits, we constructed 18 cDNA libraries for transcriptome sequencing from six sample groups, which included antennae, legs and ovipositors, both before and after odor exposure. Each group consisted of three biological replicates. We successfully obtained a total of 1253.5 million clean reads, amounting to 125.35 Gb of data (see Table S1). Each sample yielded over 6 Gb of clean data, with average Q20 and Q30 scores exceeding 96.59% and 90.84%, respectively. The average similarity rate between each sample and the reference genome was found to be 76.10% (refer to Table S1).

The box line plot depicting the distribution of FPKM (fragments per kilobase of transcript per million fragments mapped) for the samples is illustrated in Figure S1a. The variability in gene expression levels across samples was minimal, indicating a generally high level of overall gene expression. The expression density in the distribution plot suggests that gene abundance was relatively concentrated across the different tissue samples (Figure S1b). Additionally, the correlation heatmap (Figure S2) highlights a strong degree of biological reproducibility among the samples, with Pearson’s correlation coefficient (R²) exceeding 0.8 for the biological replicate samples.

#### 2.3.2. Functional Annotation Analysis of Differentially Expressed Genes

A total of 50112 Unigenes were identified in the clean data after de novo assembly.. COG, KEGG, KOG, Pfam, Swissprot, GO and NR were functionally annotated to 6862, 10,273, 10,626, 10,783, 12,957, 13,247 and 21,679 genes, respectively (Figure S3). Among the screened DEGs, there were 27 DEGs in the antennae of female *N. asiatica* after odor exposure compared with the control group (CK-HA vs. TM-HA), including 9 upregulated genes and 18 downregulated genes. Similarly, 24 DEGs (22 upregulated and 2 downregulated genes) and 2 DEGs (1 upregulated and 1 downregulated genes) were screened in the legs and ovipositor of gravid female *N. asiatica* before/after odor exposure, respectively. The number of DEGs was different among the three different tissues under odor stimulation (Table 1).

**Figure 3:**
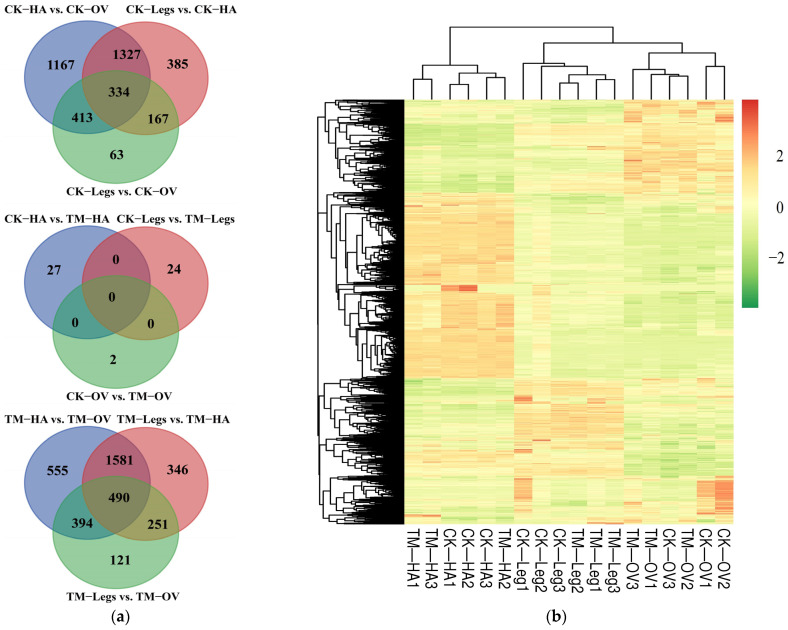
Distribution characteristics of differentially expressed genes (DEGs) in three different comparison groups. (**a**) The Venn diagram illustrates the number and proportion of DEGs that were commonly or uniquely expressed in pairwise comparisons. (**b**) A clustering analysis of DEGs’ transcript abundance across all samples is shown, with the numbers (such as the 1, 2, 3 mark to CK-HA) indicating the three biological replicates for each sample. HA: antennae; OV: ovipositor. TM represents the treatment group that was exposed to VOCs.

**Figure 4:**
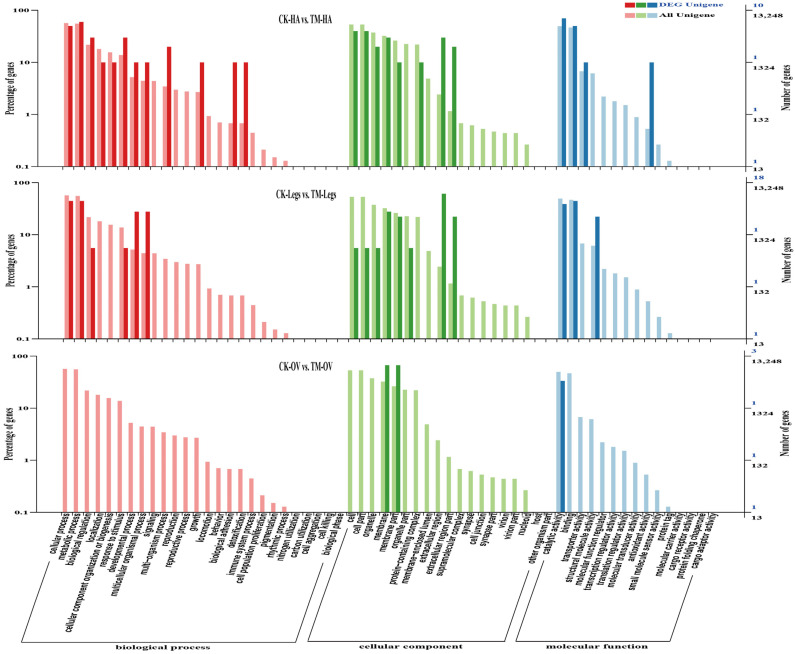
Gene ontology (GO) enrichment analysis was conducted on differentially expressed genes (DEGs) across comparison pairs. The top 10 enriched terms with highly significant *p*-values (≤0.05) for each comparison are displayed. The bar graph illustrates the false discovery rate values. The left y-axis represents the percentage of DEGs relative to all Unigenes, while the right y-axis shows the number of DEGs and total Unigenes. The x-axis indicates the GO categories.

As shown in Figure 3a, the Venn diagram showed that common and uniquely expressed genes in six comparison pairs were analyzed by two or more comparisons before/after odor exposure. A large number of common DEGs were present between CK-HA vs. CK-OV and CK-Legs vs. CK-HA or TM-HA vs. TM-OV and TM-Legs vs. TM-HA. By contrast, few common DEGs were distributed between CK-Legs vs. CK-HA and CK-Legs vs. CK-OV or TM-Legs vs. TM-HA and TM-Legs vs. TM-OV. Surprisingly, two or more comparisons among the six comparison groups detected only the uniquely expressed genes (CK-HA vs. TM-HA, CK-Legs vs. TM-Legs and CK-OV vs. TM-OV) (Figure 3a). The hierarchical clustering heatmap constructed by each sample’s FPKM expression matrix indicates the expression patterns of all DEGs across the six treatments (each treatment with three replicates). This clustering heatmap shows that some genes were highly expressed in the antennae and ovipositor of the control groups, whereas the expression levels of some genes varied significantly among CK-HA/Legs/OV and TM-HA/Legs/OV (Figure 3b). Gene ontology (GO) analysis was mainly used to classify DEG annotations among the six comparison pairs to further analyze their biological functions (Figure 4). DEG GO annotation showed a significant difference in the three different tissue parts, especially in the ovipositor, where there was no annotation of biological process categories (Figure 4).

### 2.4. Identification and Differential Expression Analysis of Chemosensory Genes After Young Goji Fruit VOC Exposure

In this study, we identified 32 candidate chemosensory genes, mainly including 28 *NasiOBPs* and 4 *NasiCSPs*, in *N. asiatica* by bioinformatics analysis (Table 2). Of these, 28 sequences had complete ORFs encoding 111 to 254 amino acids, and the remaining 4 sequences (Nasi*OBP19d2* and Nasi*OBP99a5-a7*) were partial sequences from the NCBI BLASTp analysis. Among the 32 putative olfactory proteins, 28 protein sequences were predicted to have signal peptides by SignalP 4.1 Server analysis. In addition, these sequences had BLASTp best hits to Diptera Tephritidae sequences with an e-value < 1 × 10^−5^ (Table 2).

#### 2.4.1. Identification and Expression Analysis of OBP Genes After Young Goji Fruit VOC Exposure

**Figure 5:**
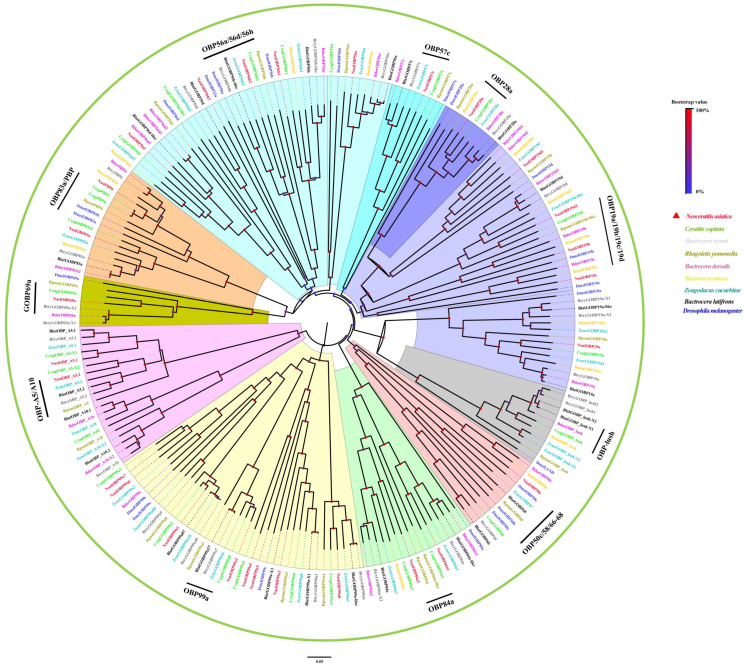
Phylogenetic tree of candidate NasiOBPs with known Dipteran OBPs. The clades in different colors indicate the different OBP gene clades. The red triangle symbol marks *Neoceratitis asiatica*. The GenBank accession numbers of these OBPs involved in the construction of this tree are listed in Table S2.

**Figure 6:**
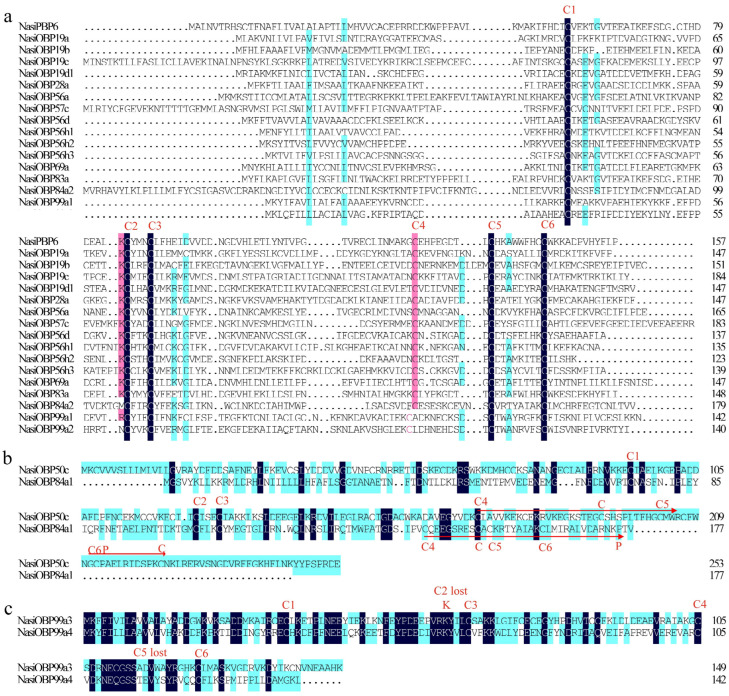
Conserved structural analysis of candidate OBPs in *N. asiatica*. (**a**) Classic OBP analysis. (**b**) Plus-C OBP analysis. (**c**) Minus-C analysis. The abbreviation “C” indicates cysteine residue. The abbreviation “P” indicates proline residue. The abbreviation “K” indicates lysine residue. The numbers 1, 2, 3, 4, 5 and 6 marked to the right of “C” indicate the six cysteine conserved structures in the sequence.

Among the 28 putative NasiOBPs, two were categorized into the PBP and OBP83a clades of Diptera within the phylogenetic tree (Figure 5), designated as NasiPBP6 and NasiOBP83a, respectively. The remaining OBPs were named according to their similarity to known Dipteran OBPs in the same tree (Figure 5). Analyzing the cysteine conserved motif patterns in the protein sequences revealed that the full-length NasiOBPs could primarily be classified into three groups: classic-C OBPs, which include NasiPBP6, OBP19a-d1, 28a, 56a, 56d, 56h1-h3, 57c, 69a, 83a, 84a2 and 99a1, all characterized by a conserved pattern of six cysteine residues (C1−X23−29−C2−X3−C3−X31−41−C4−X8−12−C5−X8−C6) (Figure 6a); plus-C OBPs, which consist of NasiOBP50c and NasiOBP84a1, featuring one or two additional cysteine residues along with a distinctive proline (P) while still maintaining the conserved six cysteine pattern (Figure 6b); and minus-C OBPs, represented by NasiOBP99a3 and NasiOBP99a4, which have lost cysteine residues C2 and C5 (Figure 6c).

**Figure 7:**
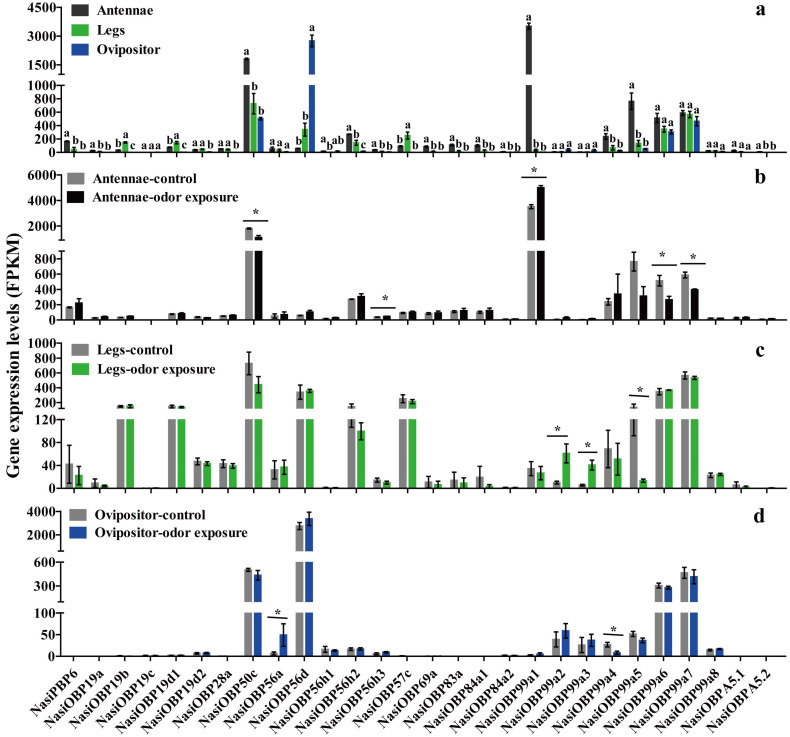
The expression levels of 28 candidate *NasiOBPs* in the antennae, legs and ovipositor of female *N. asiatica* following odor exposure are presented as FPKM values from transcriptome data. The control group consisted of female adults that were not exposed to odors. Odor sources were obtained from the volatiles of young wolfberry fruits (green fruits, 5–7 days after flower drop). (**a**) *OBP* expression levels (FPKM) in the antennae, legs and ovipositor of female *N. asiatica*. (**b**) *OBP* expression levels (FPKM) in the antennae of female *N. asiatica* before/after odor exposure. (**c**) *OBP* expression levels (FPKM) in the legs of female *N. asiatica* before/after odor exposure. (**d**) *OBP* expression levels (FPKM) in the ovipositor of female *N. asiatica* before/after odor exposure. Bars labeled with different letters (a,b,c) indicate significant differences (mean ± SE, *n* = 3, Tukey’s HSD, *p* < 0.05). An asterisk (*) denotes a significant difference between the control and odor exposure groups (mean ± SE, *n* = 3, Student’s *t*-test, * *p* < 0.05).

The results of RNA-Seq show that most OBP genes had higher FPKM values in the antennae than in the legs and ovipositor of female *N. asiatica* (*p* < 0.05), especially *NasiOBP99a1* with specific expression in the antennae (Figure 7a). After odor exposure, the transcriptome data showed that the expression of five *NasiOBPs* (*OBP50c*, -*99a6* and *-99a7* in the antennae, *OBP99a5* in the legs and *OBP99a4* in the ovipositor) was significantly downregulated, while the expression of five *NasiOBPs* (*OBP56h3* and *-99a1* in the antennae, *OBP99a2* and *-99a3* in the legs and *OBP56a* in the ovipositor) was significantly upregulated in the antennae, legs and ovipositor of female *N. asiatica* in comparison with the control (Figure 7b–d).

#### 2.4.2. Identification and Expression Analysis of CSP Genes After Young Goji Fruit VOC Exposure

**Figure 8:**
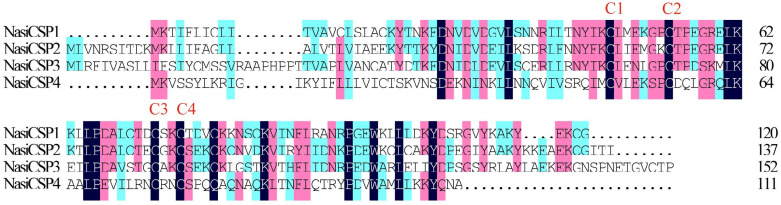
Conserved structural analysis of candidate CSPs in *N. asiatica*. The abbreviation “C” indicates cysteine residue. C1-X6-C2-X18-C3-X2-C4 indicates four cysteine conserved structures.

**Figure 9:**
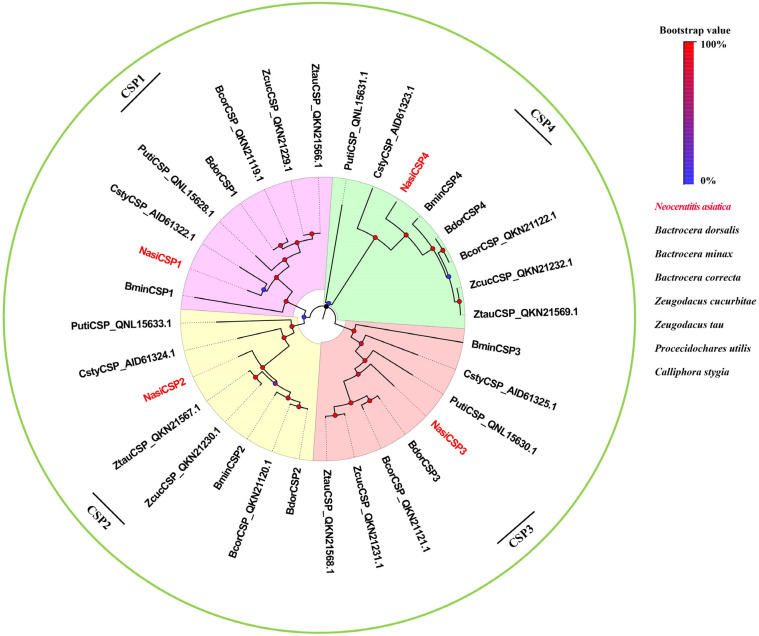
Phylogenetic tree of candidate NasiCSPs with known Dipteran CSPs. The clades in different colors indicate the different CSP gene clades. The GenBank accession numbers of these CSPs involved in the construction of this tree are listed in Table S2.

**Figure 10:**
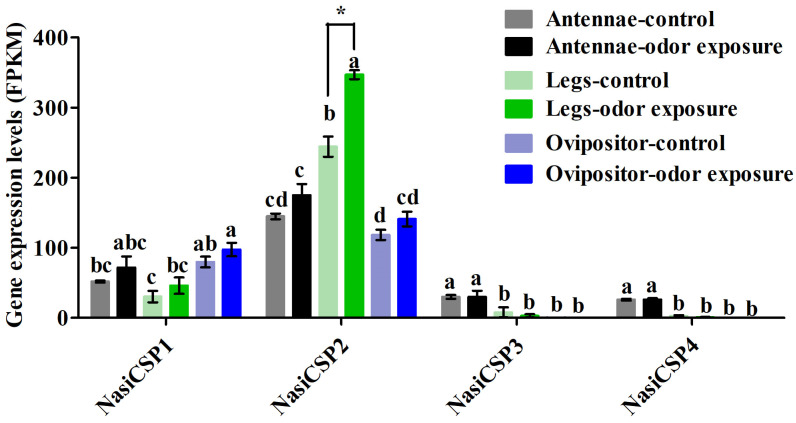
Expression levels of 4 candidate *NasiCSPs* in the antennae, legs and ovipositor of female *N. asiatica* following odor exposure are represented by FPKM values from transcriptome data. The control group consisted of female adults that were not exposed to any odors. Odor sources were derived from the volatiles of young goji fruits (green fruits, 5–7 days post-flower drop). An asterisk (*) indicates a significant difference between the control and odor exposure groups (mean ± SE, *n* = 3, Student’s *t*-test, * *p* < 0.05). Bars marked with different letters (a,b,c,d) represent significant differences among groups (mean ± SE, *n* = 3, Tukey’s HSD, *p* < 0.05).

Four sequences were identified and named NasiCSP1-4 on the basic of the similarity to known Dipteran CSPs (Table 2). The conserved cysteine pattern of C_1_-X_6_-C_2_-X18-C3-X_2_-C_4_ was present in the protein sequences of *N. asiatica* CSPs (Figure 8). As shown in Figure 9, NasiCSP1-4 were clustered into the different CSP clades of Diptera in the phylogenetic tree. The results of RNA-Seq based on the FPKM values showed that the expression levels of *NasiCSP3* and *NasiCSP4* in the antennae were significantly higher than in the legs and ovipositor of female *N. asiatica*; however, *NasiCSP2* expression was prominently higher in the legs (Figure 10). In addition, only *NasiCSP2* expression changed significantly and was upregulated in the legs after odor exposure (Figure 10).

### 2.5. RT-qPCR Verification of OBP and CSP Genes After Young Goji Fruit VOC Exposure

**Figure 11:**
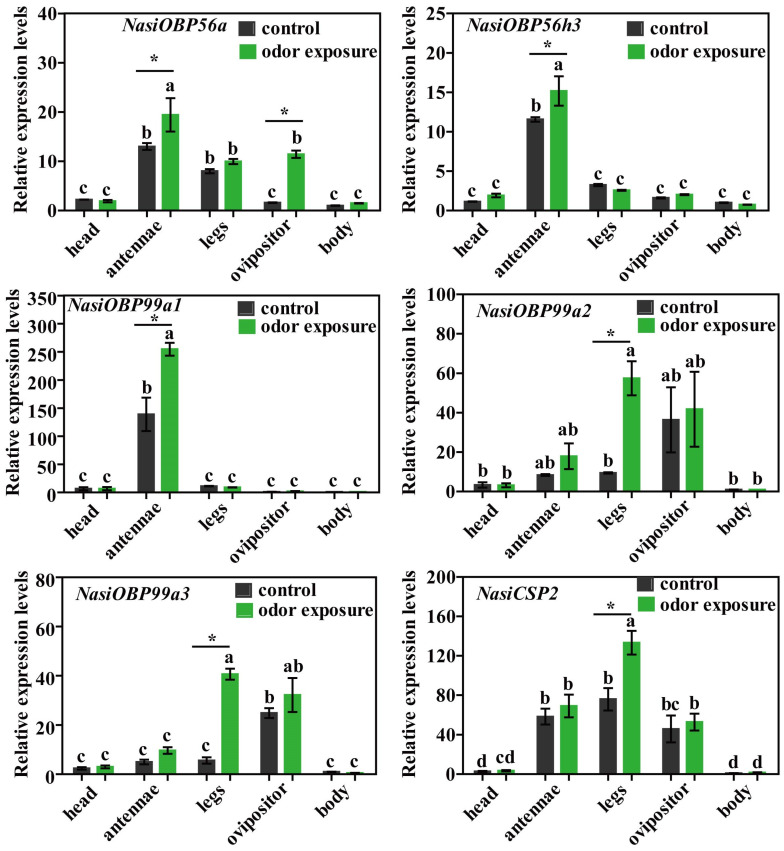
The expression profiles of six upregulated olfactory genes in different tissues of gravid female *N. asiatica* by RT-qPCR. Head indicates the head of *N. asiatica* female adult without antennae. Odor sources were obtained from the volatiles of young goji fruits (green fruits, 5–7 days post-flower drop). Body indicates a mixture of tissues from female *N. asiatica*, including thorax, abdomen and wings. Expression levels of each gene in the body (control) were used for normalization, with relative expression levels shown as fold changes compared with the transcript levels in the body (control). Data are represented as the means ± SEs. Bars with different letters indicate significant differences (*p* < 0.05, ANOVA followed by Tukey’s HSD test, *n* = 3). An asterisk (*) denotes a significant difference between control and odor exposure groups (mean ± SE, *n* = 3, Student’s *t*-test, * *p* < 0.05).

To verify the expression result of Illumina sequencing, six upregulated genes (*NasiOBP56a*, *OBP56h3*, *OBP99a1*-*a3* and *CSP2*) after odor exposure were selected, and their expression levels were detected by qPCR analysis. The qPCR results show that the expression levels of the six selected upregulated genes after exposure to host plant volatiles were mostly consistent with the results of RNA-Seq (Figure 11). Most notably, *NasiOBP99a1* and *NasiCSP2* were predominantly expressed in the antennae and legs, respectively. However, *NasiOBP56a* expression in the antennae between the results of qPCR and RNA-Seq had obvious differences. *NasiOBP56a* expression was significantly upregulated in the antennae after odor exposure according to qPCR analysis (Figure 11), while there was no difference based on Illumina sequencing analysis (Figure 7). These differences in the results need further research for confirmation.

## 3. Discussion

As people pay more and more attention to health issues, goji berries are gradually coming into people’s view, and eating and soaking goji berries has become a part of daily health. Its fruits are also popular with insects such as aphids and the goji fruit fly for their high nutrient content during the growth of goji berry plants [3]. Once goji fruits are infested by goji fruit flies, the yield and quality of goji berries are greatly reduced [7]. However, there are currently no very effective techniques to control goji fruit flies. In addition, the mechanisms underlying the host selection of *N. asiatica* for oviposition are still unclear. Therefore, it is important to clarify the oviposition selection mechanism of the goji fruit flies in order to explore green and efficient control techniques. The present study focused on the effects of goji fruit VOCs on oviposition behavior and chemosensory gene expression in *N. asiatica*.

The role of odorant-binding proteins (OBPs) in the olfactory processes of insects is well documented, particularly in their involvement in behaviors critical for survival and reproduction, such as foraging and mating. In our study, we identified 28 candidate *NasiOBPs* in *N. asiatica* through bioinformatics analysis. This finding has expanded our understanding of OBP diversity in various Tephritidae species, such as the identification of 30 OBPs in *Bactrocera dorsalis* and 33 OBPs in *Bactrocera minax* [26,27] and the discovery of distinct OBP functions in male and female *B. dorsalis* [28]. Furthermore, the expression profiles of *NasiOBPs* showed significantly higher levels in the antennae compared with other tissues, corroborating the established role of OBPs in olfactory perception [29,30]. These results highlight the potential for NasiOBPs to play crucial roles in host plant recognition, similar to the functions observed in *B. dorsalis*, *Anastrepha fraterculus* and other related species [31,32]. Additionally, our study demonstrated that specific *NasiOBPs* exhibited differential expression patterns in response to odor exposure, with five genes showing significant upregulation. This dynamic regulation of OBP expression in response to environmental stimuli has been previously observed in *B. dorsalis*, where various OBPs were found to be influenced by attractive protein baits and volatile compounds [29,33]. The selective binding affinities of OBPs, such as those demonstrated for BdorOBP69a with methyl eugenol [31], underscore the functional specialization of these proteins in chemical communication. Our findings also align with the notion that OBPs can have varied roles beyond olfaction, as indicated by the expression of several OBPs in non-olfactory tissues [26,27]. Overall, the insights gained from our study contribute to the growing body of evidence regarding the functional diversity of OBPs across insect taxa and emphasize their significance in understanding the molecular mechanisms underlying chemosensation, which may inform future pest management strategies [32,34,35].

In recent studies, the role of CSPs in various insect species has garnered significant attention, particularly concerning their involvement in olfactory detection and behavior. For instance, in the citrus fruit fly, *B. minax*, four CSP genes (BminCSP1-4) have been identified, which exhibit typical features of the CSP family, such as an N-terminal signal peptide and conserved cysteine residues. Phylogenetic analysis suggests that these CSPs may have evolved from ancestral CSP genes, indicating their potential functional diversification within *B. minax* [36]. Similarly, in the melon fly, *Bactrocera cucurbitae*, next-generation sequencing has revealed a comprehensive array of chemosensory genes, including one CSP, alongside various odorant-binding proteins and receptors. Notably, high expression levels of specific OBPs in both male and female antennae suggest their crucial role in olfactory signaling [37]. This aligns with findings in *B. dorsalis*, where CSPs were screened alongside other olfactory-related proteins, emphasizing their importance in the olfactory system and behavioral responses to host volatiles [38]. In our study, we identified four CSPs in *N. asiatica* and observed a conserved cysteine pattern characteristic of CSPs. Notably, NasiCSP3 and NasiCSP4 exhibited significantly higher expression levels in the antennae compared with other tissues, paralleling the findings in *B. minax*, where CSPs were also predominantly expressed in the antennae [36]. Moreover, the upregulation of NasiCSP2 after odor exposure suggests a dynamic response to environmental cues, akin to the expression patterns observed in *B. cucurbitae* and *B. dorsalis* [37,38]. The collective evidence from these studies highlights the critical functions of CSPs in mediating olfactory perception in various Dipteran species. The identification of candidate CSPs in our research not only contributes to the understanding of chemosensory mechanisms in *N. asiatica* but also underscores the evolutionary conservation and functional significance of CSPs across different insect taxa. This knowledge may pave the way for future investigations aimed at developing targeted pest management strategies, leveraging the olfactory pathways mediated by CSPs [39,40].

Plant volatiles modulate host localization and egg-laying behaviors in insects, whose highly sensitive olfactory system recognizes these chemical signals for behavioral responses [8,19]. In the current study, we analyzed the transcriptome of antennae, legs and ovipositor from gravid female *N. asiatica* and identified 32 soluble olfactory proteins, including 28 OBPs and 4 CSPs. Compared with the antennal transcriptomes in Diptera species from *B. dorsalis* (30 OBPs and 4 CSPs) [41,42], *B. minax* (33 OBPs and 4 CSPs) [43], *B. cucurbitae* (35 OBPs and 1 CSPs) [37] and *Bactrocera correcta* (34 OBP and 4 CSPs) [44], our NasiOBPs has no notable difference in the identified gene numbers. Exposure to volatile chemicals alters the expression levels of insects’ *OBPs*, odorant receptors (*ORs*) and odorant-degrading enzymes (*ODEs*) [45,46,47]. Further studies have indicated that these upregulated genes are involved in the process of odorant recognition [44,48]. This research study showed that the transcription expression levels of three genes (*NasiOBP56a, NasiOBP56h3* and *NasiOBP99a1*) in the antennae, three genes (*NasiOBP99a2*, *NasiOBP99a3* and *NasiCSP2*) in the legs and one gene (*NasiOBP56a*) in the ovipositor increased significantly after gravid female *N. asiatica* were exposed to goji fruit VOCs. A similar expression pattern of OBPs in the antennae was also observed in *Holotrichia oblita* and *Grapholita molesta* [44,49]. These results indicate that six upregulated genes may be involved in the recognition of young goji fruit volatiles by gravid female *N. asiatica*. *NasiOBP56a* expression was significantly upregulated not only in the antennae but also in the ovipositor after odor stimulation, which may also be involved in the oviposition selection process of gravid female *N. asiatica*. Some studies revealed the role of Drosophila melanogaster OBP56a in response to benzaldehyde [50] and *Phormia regina* OBP56a solubilizing fatty acids during feeding [51] and subsequently found that the OBP56a gene is highly expressed in female flies of *Anastrepha obliqua* [52] and *N. asiatica* [7], but no studies have shown that OBP56a is involved in the oviposition selection process of insects. Our study lacks the validation of gene function, and further work will be carried out to confirm the function of OBP56a. Here, we highlight that stimulation with goji fruit volatiles modulates the expression of olfactory genes in gravid female *N. asiatica.*

In conclusion, this study clarified for the first time the role of young goji fruit volatiles in the regulation of oviposition behavior and their effects on the expression of olfactory genes in gravid female *N. asiatica*. We concluded that the six upregulated genes in *N. asiatica* after odor stimulation may be involved in the recognition of young goji fruit volatiles. This research study focused on the ability of young goji fruit volatiles to regulate the oviposition behavior of *N. asiatica* and provides guidance for further research on the development of goji fruit fly attractants. Furthermore, this study lays a foundation for the future elucidation of the oviposition selection mechanism in gravid female *N. asiatica*.

## 4. Materials and Methods

### 4.1. Materials and Sample Preparation

#### 4.1.1. Insect Rearing and Sample Preparation

*N. asiatica* pupae were collected in Zhongning City, Ningxia Hui Autonomous Region, China, between 15 and 22 June 2023. The collected pupae were then kept at the Institute of Medicinal Plant Development, Chinese Academy of Medical Sciences, and Peking Union Medical College. After eclosion, the adult insects (with a male-to-female ratio of 1:1) were provided with a diet of 10% honey water and housed in a gauze cage (50 cm× 50 cm × 50 cm) within an artificial climate cabinet (PXZ-430B; Ningbo Jiangnan Instrument Factory, Ningbo, China). The conditions in the cabinet included a photoperiod of 14 h of light and 10 h of darkness, maintained at a temperature of 25 ± 2 °C and 40% relative humidity (RH). Four days post-eclosion, the mated female adults were collected for transcriptome sequencing and RT-qPCR validation.

#### 4.1.2. Wolfberry Cultivation and Sample Preparation

Wolfberry seedlings (Ningqi No. 5, three years old) were purchased from the Qixin wolfberry seedlings professional cooperative in Zhongning country in May 2021. Then, these wolfberry seedlings were planted at a Beijing experimental base. The goji fruits 5–7 days after flower drop were collected for odorant collection and oviposition experiments.

### 4.2. Oviposition Behavior Response of Gravid Female N. asiatica to Young Goji Fruit Volatiles

#### 4.2.1. Collection of Volatile Organic Compounds (VOCs) from Fresh Young Goji Fruits

A push–pull system was employed to capture headspace volatile organic compounds (VOCs), following the methodology outlined in a previous study [43]. In summary, approximately 500 g of fresh young goji fruits (5–7 days post-flower drop) was placed in a 1000 mL glass jar for extraction. Air was drawn through a vacuum pump (Qianxi Air Company, Beijing, China) and passed through an activated charcoal filter, followed by a sorbent cartridge (Porapak Q; 50 mg, 80/100 mesh; Supelco, Bellefonte, PA, USA) at a flow rate of 300 mL/min. The sorbent material was contained between plugs of glass wool within a glass tube measuring 10 cm in length and 0.5 cm in inner diameter. Samples were collected under controlled conditions of 25 ± 2 °C and 40% relative humidity (RH) over a period of 8 h. The odor volatiles were subsequently eluted by using 500 μL of hexane (HPLC grade, Sigma-Aldrich (Shanghai) Trading Co., Ltd., Shanghai, China) at room temperature. The resulting sample volumes were then concentrated to 200 μL by passing a slow stream of nitrogen. This experiment included three biological replicates.

#### 4.2.2. Behavioral Assays by Y-Tube Olfactometer

Behavioral assays were conducted by using a glass Y-tube olfactometer, which features a body length of 10 cm, an arm length of 8 cm, an inner diameter of 1.5 cm and an angle of 60 degrees between the two arms [47]. Air was drawn through a vacuum pump (Qianxi Air Company, Beijing, China) and filtered via an activated charcoal filter and a sorbent cartridge (Porapak Q; 50 mg, 80/100 mesh; Supelco, Bellefonte, PA, USA) at a flow rate of 300 mL/min. Experimental samples consisting of volatile organic compounds (VOCs) from young goji fruits were placed in one arm, while control samples of HPLC-grade hexane were placed in the other arm. Individual mated female N. asiatica flies, four days post-eclosion, were introduced at the entrance of the olfactometer. The number of female flies that entered each arm and remained for at least 30 s was recorded within a 5 min observation period. Flies that did not make a choice within this timeframe were not counted. After each trial involving five flies, the airflow direction was alternated between the arms. A total of 28 adult flies were tested in each experiment by using the Y-tube olfactometer. All assays were conducted in a controlled behavior room maintained at 25 ± 2 °C and 40% relative humidity (RH). The olfactometer was thoroughly washed and dried prior to each experiment, and each experimental setup was replicated three times.

#### 4.2.3. Comparison of Oviposition on Agar Medium with and Without Goji Young Fruits VOCs

Two media (containing 10% agar) were placed on the opposite corners of an insect cage (length of 50 cm × width of 50 cm × height of 50 cm). One medium had a filter paper strip (length of 2 cm × width of 1 cm) with 10 μL of goji fruit VOCs, and the other medium had a filter paper strip (length of 2 cm × width of 1 cm) with 10 μL of paraffin oil (control). The 30 gravid female *N. asiatica* were released in the center of this insect cage. After 24 h, the numbers of eggs laid by *N. asiatica* were counted on the media under a stereoscope (EZ4W; Leica Microsystems AGCH-9435 Heerbrugg, Schweiz). Three biological replicates were set in this experiment.

### 4.3. Transcriptome Analysis of Female N. asiatica Antennae, Legs and Ovipositor Under Young Goji Fruit Volatile Exposure

#### 4.3.1. Sample Collection and RNA Extraction

The mated female adults 4 days after eclosion were collected before (control)/after (treatment) goji fruit (5–7 days after flowers) volatile exposure for transcriptome sequencing. Volatiles from goji fruits were absorbed onto filter paper, which was subsequently positioned in the center of a plexiglass cage (50 × 50 × 50 cm). Test female adults were then released into the plexiglass cage. For the control group, test female adults were placed in a separate plexiglass cage devoid of volatiles. After a duration of 2 h, various tissue samples were collected and rapidly frozen in liquid nitrogen for further analysis.

The tissue parts for sequencing included 300 pairs of antennae, 180 legs (selected 30 female flies) and 300 ovipositors from 300 female flies for each experiment. These samples were immediately frozen and stored in liquid nitrogen until RNA extraction. Three biological replicates were set in this experiment. The total RNA of the samples was extracted by using Trizol reagent (Invitrogen, Shanghai, China). RNA quantity was determined by using a NanoDrop 2000 spectrophotometer (NanoDrop, Wilmington, DE, USA). Before sequencing, the RNA samples were stored at −80 °C.

#### 4.3.2. cDNA Construction, Illumina Sequencing, Assembly and Annotation

The construction of the cDNA library and the subsequent Illumina sequencing of our RNA samples were carried out at Biomarker Technologies Co., Ltd., located in Beijing, China. Initially, the purity, concentration and integrity of each RNA sample (2 μg) were assessed by using the NanoDrop 2000, Qubit 2.0 (Invitrogen, Carlsbad, CA, USA) and Agilent 2100 (Agilent Technologies, Santa Clara, CA, USA) methods. Sequencing was performed on an Illumina HiSeq™ 4500 platform, employing a paired-end (PE125) strategy that produced paired-end reads of 125 base pairs each.

High-quality clean reads were derived from the raw sequencing data by eliminating any reads that contained adapters or poly-N sequences or were of low quality. The transcriptome was then assembled de novo by using the short read assembly software Trinity v2.15.1 [53]. Following this, the outputs from Trinity were clustered by using TGICL 2.1 [54]. The resulting consensus cluster sequences, along with singletons, formed the Unigene dataset. Unigene annotation was conducted by using NCBI BLASTx against a combined database of non-redundant (nr) and Swiss-Prot protein sequences, applying an e-value threshold of <1 × 10^−5^. The results from the BLAST analysis were subsequently imported into the Blast2GO [55] pipeline for gene ontology (GO) annotation. Additionally, the prediction of protein-coding regions was carried out by using OrfPredictor [56] based on the BLAST results. The techniques and methodologies employed in this study were consistent with those used in our previous research [57].

#### 4.3.3. Identification, Phylogenetic Construction and Differentially Expressed Genes Analysis of OBPs and CSPs

The sequence analysis techniques utilized in this study were consistent with those described previously [54]. Open reading frames (ORFs) for the odorant-binding proteins (OBPs) and chemosensory proteins (CSPs) in the goji fruit fly were predicted by using the online ORF Finder tool (http://www.ncbi.nlm.nih.gov/gorf/gorf.html, accessed on 10 August 2023). Similarity searches were conducted via the NCBI-BLAST web server (http://blast.ncbi.nlm.nih.gov/, accessed on 15 August 2023). The potential N-terminal signal peptides for the OBPs and CSPs were predicted by using the SignalP 4.0 server (http://www.cbs.dtu.dk/services/SignalP/, accessed on 20 August 2023). Multiple-sequence alignments of the amino acid sequences for NasiOBPs and NasiCSPs from *N. asiatica* were carried out by using ClustalX 2.0 [58]. Phylogenetic trees were constructed with MEGA 11, employing the maximum likelihood method based on a p-distance model and utilizing pairwise deletion for gaps. Bootstrap support was evaluated through a bootstrap procedure involving 1000 replicates. The cleaned transcriptome data of *N. asiatica* have been uploaded to the Sequence Read Archive (SRA) at the National Center for Biotechnology Information (NCBI). The accession numbers are SRX18291226-SRX18291235 and SRX18291237-SRX18291244.

### 4.4. RT-qPCR Verification of OBPs and CSPs in N. asiatica

#### 4.4.1. RNA Extraction and cDNA Synthesis

RNA and first-strand cDNA from the samples collected were extracted and synthesized by using Invitrogen TRIzol Reagent (Invitrogen, Carlsbad, CA, USA) and the PrimeScript™ RT Reagent Kit with gDNA Eraser (Takara, Dalian, China), respectively. The quantity and quality of the total RNA were assessed by using a NanoDrop 2000 spectrophotometer (NanoDrop, Wilmington, DE, USA). Subsequently, 1 μg of the total RNA was utilized for cDNA synthesis.

#### 4.4.2. Primer Design, Primer Evaluation and qPCR Analysis

Following exposure to VOCs from young goji fruit, we selected six upregulated genes (*Nasi-OBP56a*, *OBP56h3*, *OBP99a1-a3* and *CSP2*) along with two reference genes (RPS13 and EF1α) for expression level analysis via qPCR [7]. Specific primers for these genes were designed by using NCBI Primer-BLAST (https://www.ncbi.nlm.nih.gov/tools/primer-blast/index.cgi?LINK_LOC=BlastHome, accessed on 15 September 2023), and the details are provided in Table S3. The synthesized cDNA was diluted in a three-fold series (1/3, 1/9, 1/27, 1/81 and 1/243) to create a standard curve. The amplification efficiency of each primer was automatically analyzed by using Bio-Rad CFX 3.0 software. The specificity of the qPCR primers was verified through melting curve analysis and the sequencing of the qPCR products [7]. The qPCR techniques and methodologies employed in this study were consistent with those used in previous research [7].

### 4.5. Statistical Analysis

All statistical analyses were conducted by using SPSS 26.0 software (SPSS Inc., Chicago, IL, USA). Multiple comparisons among data sets were evaluated by using ANOVA, followed by the Tukey’s HSD test (*p* < 0.05). Normality and homogeneity of variance tests were conducted on all data by using the Shapiro–Wilk and Bartlett tests. For assessing the statistical significance between two treatments, a pairwise Student’s *t*-test was performed (*p* < 0.05) [7].

## Figures and Tables

**Table 1:** DEGs in the different treatment groups.

Sample Comparisons	Total No. of Significantly DEGs	Total No. of Significantly Upregulated DEGs	Total No. of Significantly Downregulated DEGs
CK-HA vs. TM-HA	27	9	18
CK-HA vs. CK-OV	3241	1413	1828
CK-Legs vs. CK-HA	2213	995	1218
CK-Legs vs. TM-Legs	24	22	2
CK-Legs vs. CK-OV	977	334	643
CK-OV vs. TM-OV	2	1	1
TM-HA vs. TM-OV	3020	1414	1606
TM-Legs vs. TM-HA	2668	1370	1298
TM-Legs vs. TM-OV	1256	546	710

Note: DEGs, differentially expressed genes. No., number. CK-HA: healthy antennae control of female *N. asiatica* before odor exposure. TM-HA: healthy antennae treatment of female *N. asiatica* after odor exposure. CK-Legs: legs control of female *N. asiatica* before odor exposure. TM-Legs: legs treatment of female *N. asiatica* after odor exposure. CK-OV: ovipositor control of female *N. asiatica* before odor exposure. TM-OV: ovipositor treatment of female *N. asiatica* after odor exposure.

**Table 2:** Best BLAST hits for candidate chemosensory genes identified in transcriptome database of *N. asiatica* adults.

Gene Name	Length (nt)	ORF (aa)	Complete ORF	Signal Peptide	BLASTp Best Hit			
					BLAST Protein Name	Identify (%)	E-Value	Acc. Number
NasiPBP6	1741	157	Y	Y	pheromone-binding protein-related protein 6 [*Ceratitis capitata*]	94.27	2.00 × 10^−108^	XP_020713726.1
NasiOBP19a	1125	147	Y	Y	general odorant-binding protein 19a [*Ceratitis capitata*]	86.39	1.00 × 10^−89^	XP_004525026.1
NasiOBP19b	1408	151	Y	Y	odorant-binding protein 19b [*Bactrocera minax*]	60.93	3.00 × 10^−57^	AYN70630.1
NasiOBP19c	1659	184	Y	Y	odorant-binding protein 19c [*Bactrocera minax*]	73.37	7.00 × 10^−94^	AYN70631.1
NasiOBP19d1	4676	147	Y	Y	general odorant-binding protein 19d [*Ceratitis capitata*]	88.73	9.00 × 10^−88^	XP_004525035.1
NasiOBP19d2	1236	140	N	N	general odorant-binding protein 19d [*Ceratitis capitata*]	82.73	2.00 × 10^−79^	XP_004525139.2
NasiOBP28a	931	147	Y	Y	general odorant-binding protein 28a [*Ceratitis capitata*]	89.8	3.00 × 10^−91^	XP_004525016.1
NasiOBP50c	1074	254	Y	Y	odorant-binding protein 50c [*Bactrocera minax*]	73.75	6.00 × 10^−128^	AYN70637.1
NasiOBP56a	697	165	Y	Y	general odorant-binding protein 56a [*Ceratitis capitata*]	87.88	2.00 × 10^−102^	XP_020718149.1
NasiOBP56d	815	137	Y	Y	general odorant-binding protein 56d [*Ceratitis capitata*]	78.20	8.00 × 10^−61^	XP_004517803.1
NasiOBP56h1	1344	135	Y	Y	general odorant-binding protein 56h [*Ceratitis capitata*]	77.94	8.00 × 10^−73^	XP_004517804.1
NasiOBP56h2	577	123	Y	Y	general odorant-binding protein 56h-like [*Ceratitis capitata*]	72.58	7.00 × 10^−66^	XP_004518466.2
NasiOBP56h3	604	139	Y	Y	general odorant-binding protein 56h-like [*Bactrocera oleae*]	56.31	2.00 × 10^−33^	XP_014102006.2
NasiOBP57c	1544	184	Y	N	odorant-binding protein 57c [*Bactrocera dorsalis*]	64.13	4.00 × 10^−76^	AKI29013.1
NasiOBP69a	776	147	Y	Y	general odorant-binding protein 69a precursor [*Ceratitis capitata*]	84.35	2.00 × 10^−89^	NP_001295335.1
NasiOBP83a	1039	148	Y	Y	general odorant-binding protein 83a precursor [*Ceratitis capitata*]	91.22	6.00 × 10^−95^	NP_001295333.1
NasiOBP84a1	984	177	Y	Y	general odorant-binding protein 84a [*Ceratitis capitata*]	83.71	4.00 × 10^−106^	XP_012158643.1
NasiOBP84a2	951	179	Y	Y	general odorant-binding protein 84a [*Ceratitis capitata*]	74.86	2.00 × 10^−92^	XP_004529369.1
NasiOBP99a1	961	142	Y	Y	general odorant-binding protein 99a [*Ceratitis capitata*]	97.89	1.00 × 10^−96^	XP_004535942.1
NasiOBP99a2	1056	140	Y	Y	general odorant-binding protein 99a [*Ceratitis capitata*]	94.29	2.00 × 10^−96^	XP_004523508.1
NasiOBP99a3	1170	150	Y	Y	general odorant-binding protein 99a [*Ceratitis capitata*]	77.40	4.00 × 10^−80^	XP_004521186.1
NasiOBP99a4	589	142	Y	Y	general odorant-binding protein 99a-like [*Ceratitis capitata*]	56.34	2.00 × 10^−44^	XP_020717484.1
NasiOBP99a5	1095	115	N	N	general odorant-binding protein 99a-like [*Bactrocera tryoni*]	55.67	6.00 × 10^−28^	XP_039959060.1
NasiOBP99a6	510	119	N	Y	general odorant-binding protein 99a [*Ceratitis capitata*]	83.67	5.00 × 10^−53^	XP_004521183.1
NasiOBP99a7	371	71	N	N	general odorant-binding protein 99a [*Ceratitis capitata*]	86.79	7.00 × 10^−25^	XP_004521183.1
NasiOBP99a8	2503	162	Y	Y	general odorant-binding protein 99a [*Ceratitis capitata*]	90.74	3.00 × 10^−109^	XP_004521185.1
NasiOBP A5.1	887	206	Y	Y	putative odorant-binding protein A5 [*Ceratitis capitata*]	92.72	1.00 × 10^−138^	XP_004531311.1
NasiOBP A5.2	752	207	Y	Y	putative odorant-binding protein A5 [*Ceratitis capitata*]	85.99	2.00 × 10^−133^	XP_004531312.1
NasiCSP1	904	120	Y	Y	chemosensory protein 3 [*Bactrocera minax*]	85.12	2.00 × 10^−67^	QOC63332.1
NasiCSP2	3619	137	Y	Y	chemosensory protein 2 [*Bactrocera minax*]	90.55	8.00 × 10^−79^	AYN70622.1
NasiCSP3	2657	153	Y	Y	chemosensory protein 3 [*Bactrocera dorsalis*]	81.12	4.00 × 10^−83^	AKI28977.1
NasiCSP4	3186	111	Y	Y	chemosensory protein 4 [*Bactrocera minax*]	87.27	1.00 × 10^−61^	AYN70623.1

## Data Availability

All data in this study will be available from the corresponding author upon reasonable request.

## Supplementary Materials

The following supporting information can be downloaded at: https://www.mdpi.com/article/10.3390/ijms252413249/s1.
