# Supplementary material for: Young Goji Fruit Volatiles Regulate the Oviposition Behavior and Chemosensory Gene Expression of Gravid Female Neoceratitis asiatica
Source: Int J Mol Sci. 2024 Dec 10;25(24):13249. doi: 10.3390/ijms252413249 (PMC11675652; doi:10.3390/ijms252413249)
Supplement: Supplementary file 1 [file ijms-25-13249-s001.zip › ijms-3296463-supplementary.pdf]

Young Goji Fruit Volatiles Regulate the Oviposition Behavior and Chemosensory Gene Expression of Gravid Female *Neoceratitis asiatica*

Hongshuang Wei [1], Kexin Liu [1], Jingyi Zhang [1], Kun Guo [1], Sai Liu [1], Changqing Xu [1], Haili Qiao [1,*] and Shuqian Tan [2,*]

[1] State Key Laboratory for Quality Ensurance and Sustainable Use of Dao-di Herbs, Institute of Medicinal Plant Development, Chinese Academy of Medical Sciences, Peking Union Medical College, Beijing 100193, China; hswei@implad.ac.cn (H.W.); liukexin202@163.com (K.L.); zhongyaoxili@163.com (J.Z.); kguo@implad.ac.cn (K.G.); sliu@implad.ac.cn (S.L.); cqxu@implad.ac.cn (C.X.)

[2] Key Lab of Integrated Pest Management, Department of Entomology, College of Plant Protection, China Agricultural University, Beijing 100193, China

* Correspondence: hlqiao@implad.ac.cn (H.Q.); sqtan@cau.edu.cn (S.T.)

Table S1. Summary of sequencing data of all samples.

Sample	Clean reads	Clean bases	Q20 (%)	Q30 (%)	GC content	mapped reads	Mapped ratio
CK—HA1	21829033	6.71	96.62	91.47	35.41	16091963	73.72%
CK—HA2	23658495	7.24	96.97	91.69	35.00	17811388	75.29%
CK—HA3	22226380	6.80	97.02	91.85	36.43	16687882	75.08%
CK—OV1	23025961	7.04	96.9	91.54	35.59	17616428	76.51%
CK—OV2	22443461	6.85	97.09	91.94	38.77	17554354	78.22%
CK—OV3	22783292	6.95	96.95	91.63	37.45	17658871	77.51%
CK—Leg1	23008807	7.02	97.11	92.02	36.45	17505204	76.08%
CK—Leg2	22592123	6.92	97.07	91.89	35.05	17277440	76.48%
CK—Leg3	23383523	7.14	97.12	91.97	35.89	17946509	76.75%
TM—HA1	23345068	7.09	97.28	92.33	36.04	18314210	78.45%
TM—HA2	21938787	6.71	96.89	91.44	34.59	16202696	73.85%
TM—HA3	23450873	7.14	97.08	91.83	36.39	17940000	76.50%
TM—OV1	23164924	7.05	96.97	91.69	37.21	17566270	75.83%
TM—OV2	23082633	7.02	97.16	92.02	38.51	17944001	77.74%
TM—OV3	23293400	7.09	97.18	92.1	39.39	18021236	77.37%
TM—Leg1	23292604	7.09	97.03	91.82	36.64	17361536	74.54%
TM—Leg2	21083150	6.41	96.59	90.84	35.22	15867405	75.26%
TM—Leg3	23299256	7.08	97.07	91.87	36.83	15867405	74.53%

Table S2. The GenBank accession numbers of OBPs and CSPs from other insect species involved in the construction of two trees.

**Gene name**	**Organism**	**Accession number**	**Gene name**	**Accession number**
CcapGOBP lush	*Ceratitis capitata*	XP_004522281.1	CcapGOBP56h2	JAC05338.1
CcapPBP6	*Ceratitis capitata*	XP_020713726.1	CcapGOBP57c	XP_004522856.1
CcapPBP3	*Ceratitis capitata*	JAC02657.1	CcapGOBP68	XP_004537654.1
CcapOBP A10	*Ceratitis capitata*	JAB88358.1	CcapGOBP69a	CDJ79886.1
CcapOBP A5-X2	*Ceratitis capitata*	XP_004531312.1	CcapOBP83a2	CDJ79887.1
CcapOBP A5-X1	*Ceratitis capitata*	XP_004531311.1	CcapGOBP84a1	XP_012158643.1
CcapGOBP19a	*Ceratitis capitata*	XP_004525026.1	CcapGOBP84a2	XP_004529369.1
CcapGOBP19d	*Ceratitis capitata*	XP_004525139.2	CcapGOBP99a4	XP_020717484.1
CcapGOBP28a	*Ceratitis capitata*	XP_004525016.1	CcapGOBP99a2	XP_004535942.1
CcapGOBP56h1	*Ceratitis capitata*	XP_004517804.1	CcapGOBP99a8	XP_004523508.1
CcapGOBP56a	*Ceratitis capitata*	JAB84797.1	CcapGOBP99a6	XP_004521186.1
CcapGOBP56d	*Ceratitis capitata*	XP_004517904.1	CcapGOBP99a5	XP_004521185.1
CcapGOBP56d-like	*Ceratitis capitata*	XP_004517904.1	CcapGOBP99a3	XP_004521183.1
BtryPBP6	*Bactrocera tryoni*	XP_039957844.1	BtryGOBP56h	XP_039955720.1

BtryGOBP lush2	*Bactrocera tryoni*	XP_039964381.1	BtryGOBP57c	XP_039953837.1
BtryGOBP lush1	*Bactrocera tryoni*	XP_039964380.1	BtryGOBP66	XP_039953053.1
BtryOBP A10	*Bactrocera tryoni*	XP_039962771.1	BtryGOBP69a-X2	XP_039966261.1
BtryOBP A5.2	*Bactrocera tryoni*	XP_039952463.1	BtryGOBP69a-X1	XP_039966260.1
BtryOBP A5.1	*Bactrocera tryoni*	XP_039952576.1	BtryGOBP83a-like	XP_039970969.1
BtryGOBP19a-X1	*Bactrocera tryoni*	XP_039958470.1	BtryGOBP84a-X2	XP_039963494.1
BtryGOBP19a-X2	*Bactrocera tryoni*	XP_039958469.1	BtryGOBP84a-like	XP_039947471.1
BtryGOBP19a	*Bactrocera tryoni*	XP_039958468.1	BtryGOBP99a3	XP_039964533.1
BtryGOBP19d-like	*Bactrocera tryoni*	XP_039960171.1	BtryGOBP99a6	XP_039948026.1
BtryGOBP28a-like	*Bactrocera tryoni*	XP_039959984.1	BtryGOBP99a9	XP_039971369.1
BtryGOBP56a-like	*Bactrocera tryoni*	XP_039955300.1	BtryGOBP99a7	XP_039971027.1
BtryGOBP56d	*Bactrocera tryoni*	XP_039955913.1	BtryGOBP99a49	XP_039956849.1
BtryGOBP56h-like	*Bactrocera tryoni*	XP_039955446.1		
RpomOBP A5	*Rhagoletis pomonella*	XP_036329576.1	RpomGOBP57c-like	XP_036334119.1
RpomOBP A10	*Rhagoletis pomonella*	XP_036319257.1	RpomGOBP68	XP_036332317.1
RpomGOBP lush	*Rhagoletis pomonella*	XP_036325672.1	RpomGOBP69a	XP_036338127.1
RpomPBP6	*Rhagoletis pomonella*	XP_036325307.1	RpomGOBP84a-like	XP_036319269.1
RpomGOBP19d	*Rhagoletis pomonella*	XP_036341363.1	RpomGOBP84a	XP_036346206.1
RpomGOBP28a-like	*Rhagoletis pomonella*	XP_036338561.1	RpomGOBP99a8	XP_036331928.1
RpomGOBP19d-like	*Rhagoletis pomonella*	XP_036339673.1	RpomGOBP99a4	XP_036324634.1
RpomGOBP56d-like	*Rhagoletis pomonella*	XP_036334579.1	RpomGOBP99a1	XP_036324631.1
RpomGOBP56h-like	*Rhagoletis pomonella*	XP_036334578.1	RpomGOBP99a3	XP_036324043.1
RpomGOBP56a-like	*Rhagoletis pomonella*	XP_036333622.1		
BdorPBP6	*Bactrocera dorsalis*	XP_019848198.1	BdorOBP56d2	AKI29010.1
BdorOBP83a2	*Bactrocera dorsalis*	AKI29016.1	BdorOBP56a	AKI29008.1
BdorOBP A10-X2	*Bactrocera dorsalis*	XP_011208559.1	BdorOBP50c	AKI29006.1
BdorOBP A10	*Bactrocera dorsalis*	JAC57212.1	BdorOBP28a	AKI29004.1
BdorOBP99a	*Bactrocera dorsalis*	AKI29022.1	BdorOBP19d4	QOE77713.1
BdorOBP84a2	*Bactrocera dorsalis*	AKI29021.1	BdorOBP19d3	AKI29003.1
BdorOBP84a1	*Bactrocera dorsalis*	AKI29020.1	BdorOBP19d1	AKI29001.1
BdorOBP69a	*Bactrocera dorsalis*	AKI29014.1	BdorOBP19c	AKI29000.1
BdorOBP56h	*Bactrocera dorsalis*	AKI29012.1	BdorOBP19b	AKI28999.1
BdorOBP57c	*Bactrocera dorsalis*	AKI29013.1	BdorOBP19a	AKI28998.1
BdorOBP56d3	*Bactrocera dorsalis*	QOE77715.1	BdorOBP lush	AKI28996.1
BdorOBP56d1	*Bactrocera dorsalis*	AKI29009.1		

BminPBP83b	*Bactrocera minax*	AYN70648.1	BminOBP19d3	AYN70634.1
BminOBP83a	*Bactrocera minax*	AYN70647.1	BminOBP19d1	AYN70632.1
BminOBP84a2	*Bactrocera minax*	AYN70651.1	BminOBP19c	AYN70631.1
BminOBP56h1	*Bactrocera minax*	AYN70643.1	BminOBP19b	AYN70630.1
BminOBP56d	*Bactrocera minax*	AYN70641.1	BminOBP19a2	AYN70629.1
BminOBP56a	*Bactrocera minax*	AYN70638.1	BminOBP19a1	AYN70628.1
BminOBP50c	*Bactrocera minax*	AYN70637.1	BminOBP lush	AYN70627.1
BminOBP28a	*Bactrocera minax*	AYN70635.1		
ZcucOBP A5.2	*Zeugodacus cucurbitae*	XP_011189397.1	ZcucGOBP56h2	XP_011180830.1
ZcucOBP A5.1	*Zeugodacus cucurbitae*	XP_011189396.1	ZcucGOBP56h1	JAD08755.1
ZcucOBP A10-X2	*Zeugodacus cucurbitae*	XP_011177223.1	ZcucGOBP57c	JAD02753.1
ZcucOBP A10	*Zeugodacus cucurbitae*	JAC99540.1	ZcucGOBP67-like	XP_011190103.1
ZcucGOBP lush-X2	*Zeugodacus cucurbitae*	XP_011176696.1	ZcucGOBP83a-like	XP_028901212.1
ZcucGOBP lush-X1	*Zeugodacus cucurbitae*	XP_011176687.1	ZcucGOBP84a2	XP_011184797.1
ZcucGOBP19d	*Zeugodacus cucurbitae*	XP_011187244.1	ZcucGOBP84a1	XP_011184805.1
ZcucGOBP19d-like	*Zeugodacus cucurbitae*	XP_011187239.1	ZcucGOBP99a10	XP_011182910.1
ZcucGOBP19a1	*Zeugodacus cucurbitae*	XP_011187213.1	ZcucGOBP99a3	XP_011184703.1
ZcucGOBP19a2	*Zeugodacus cucurbitae*	XP_011187212.1	ZcucGOBP99a1	XP_011184701.1
ZcucGOBP28a	*Zeugodacus cucurbitae*	XP_011193147.1	ZcucGOBP99a8	XP_011184698.1
ZcucGOBP56a	*Zeugodacus cucurbitae*	JAC98783.1	ZcucGOBP99a80	XP_011177680.1
ZcucGOBP56d1	*Zeugodacus cucurbitae*	JAC98207.1	ZcucGOBP99a6	XP_011191566.1
ZcucGOBP56d2	*Zeugodacus cucurbitae*	JAD02035.1		
BlatOBP A5.1	*Bactrocera latifrons*	XP_018789129.1	BlatGOBP56d	XP_018784470.1
BlatOBP A5.2	*Bactrocera latifrons*	XP_018789120.1	BlatGOBP57c	XP_018785093.1
BlatOBP A10.1	*Bactrocera latifrons*	JAI40944.1	BlatGOBP66	XP_018803987.1
BlatOBP A10.2	*Bactrocera latifrons*	JAI32118.1	BlatGOBP68	XP_018788552.1
BlatGOBP lush-X2	*Bactrocera latifrons*	XP_018790185.1	BlatGOBP83a-like	XP_018794248.1
BlatGOBP lush-X1	*Bactrocera latifrons*	XP_018790184.1	BlatGOBP84a-like	XP_018787917.1
BlatGOBP19d-like	*Bactrocera latifrons*	XP_018787263.1	BlatGOBP84a-	XP_018801147.1
BlatGOBP19a-like	*Bactrocera latifrons*	XP_018787259.1	BlatGOBP99a-X3	XP_018799093.1
BlatGOBP19a	*Bactrocera latifrons*	JAI19361.1	BlatGOBP99a-X1	XP_018799091.1
BlatGOBP28a	*Bactrocera latifrons*	XP_018787257.1	BlatGOBP99a-like	XP_018795287.1
BlatGOBP56a-like	*Bactrocera latifrons*	XP_018784478.1	BlatGOBP99a87	XP_018790487.1
BlatGOBP56h	*Bactrocera latifrons*	XP_018784477.1	BlatGOBP99a27	XP_018795827.1
BlatGOBP56h-like	*Bactrocera latifrons*	XP_018794675.1		
BdorCSP1	*Bactrocera dorsalis*	AKI28975.1	BdorCSP3	AK128977.1
BdorCSP2	*Bactrocera dorsalis*	AK128976.1	BdorCSP4	AK128978.1
BminCSP1	*Bactrocera minax*	AYN70625.1	BminCSP3	QOC63332.1
BminCSP2	*Bactrocera minax*	AYN70622.1	BminCSP4	AYN70623.1
ZcucCSP_QKN212	*Zeugodacus cucurbitae*	QKN21232.1	ZcucCSP_QKN212	QKN21230.1

32.1			30.1	
ZcucCSP_QKN21231.1	*Zeugodacus cucurbitae*	QKN21231.1	ZcucCSP_QKN21229.1	QKN21229.1
ZtauCSP_QKN21569.1	*Zeugodacus tau*	QKN21569.1	ZtauCSP_QKN21567.1	QKN21567.1
ZtauCSP_QKN21568.1	*Zeugodacus tau*	QKN21568.1	ZtauCSP_QKN21566.1	QKN21566.1
PutiCSP_QNL15633.1	*Procecidochares utilis*	QNL15633.1	PutiCSP_QNL15630.1	QNL15630.1
PutiCSP_QNL15631.1	*Procecidochares utilis*	QNL15631.1	PutiCSP_QNL15628.1	QNL15628.1
BcorCSP_QKN21122.1	*Bactrocera correcta*	QKN21122.1	BcorCSP_QKN21120.1	QKN21120.1
BcorCSP_QKN21121.1	*Bactrocera correcta*	QKN21121.1	BcorCSP_QKN21119.1	QKN21119.1
CstyCSP_AID61325.1	*Calliphora stygia*	AID61325.1	CstyCSP_AID61323.1	AID61323.1
CstyCSP_AID61324.1	*Calliphora stygia*	AID61324.1	CstyCSP_AID61322.1	AID61322.1

Table S3. Primer sequences of the selected olfactory genes and reference genes.

**Gene name**	**Primer sequence (from 5' to 3')**
**Targe gene:**	
NasiOBP56a	F: CTGAAACACGCCAAGGAAGC
	R: CGTTGGGATTGGCGACCTTA
NasiOBP56h3	F:ACAAGGAAGCCGGTGTTAC
	R:ACTGACACTTAATGGGTTCGG
NasiOBP99a1	F:AAGGAGAATAAGGATGCGGTG
	R:TGAAACCACGATAAGCCCATG
NasiOBP99a2	F:TGTATAGCATTGGTCGCAGG
	R:TCATAAATGTCATCGGGTATACGG
NasiOBP99a3	F:ATCCCGATGAAGAGCCTGTG
	R:GGTAACGTGGTCGGGATGAT
NasiCSP2	F: GTCTGATTGAAATGGGAAAGTGC
	R: TCACTACATTTGCCACACTCG
**Reference genes:**	
RPS13	F: CAAGCATTTGGAGCGTAATCG
	R: AGCGGTACTGGATTCGTATTTC
EF1α	F: GCCCAGGTTATTGTGTTGAAC
	R: GGGATTCTCTTCAGTGGTCTTAC

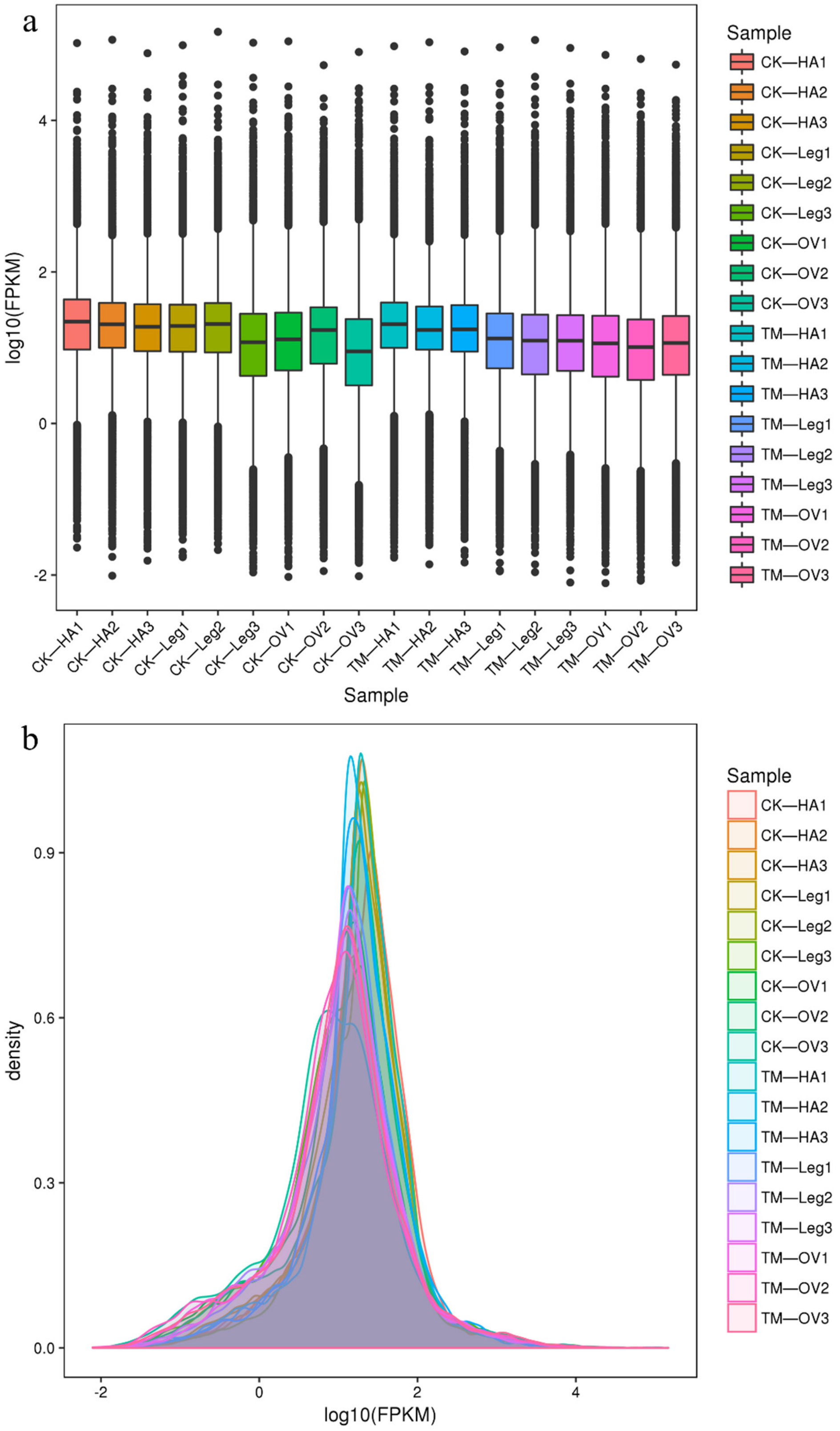

Figure S1. (a) Gene expression box line plot. Abscissa indicates different samples. Ordinate indicates logarithmic values of sample expression FPKM. The graph measures the distribution of overall gene expression levels for each

sample. (b) Expression density distribution plot. Different colors of curves in the graph represent different samples. Abscissa of points on the curve indicates logarithmic values of FPKM for corresponding samples. Ordinate of points on the curve indicates probability density.

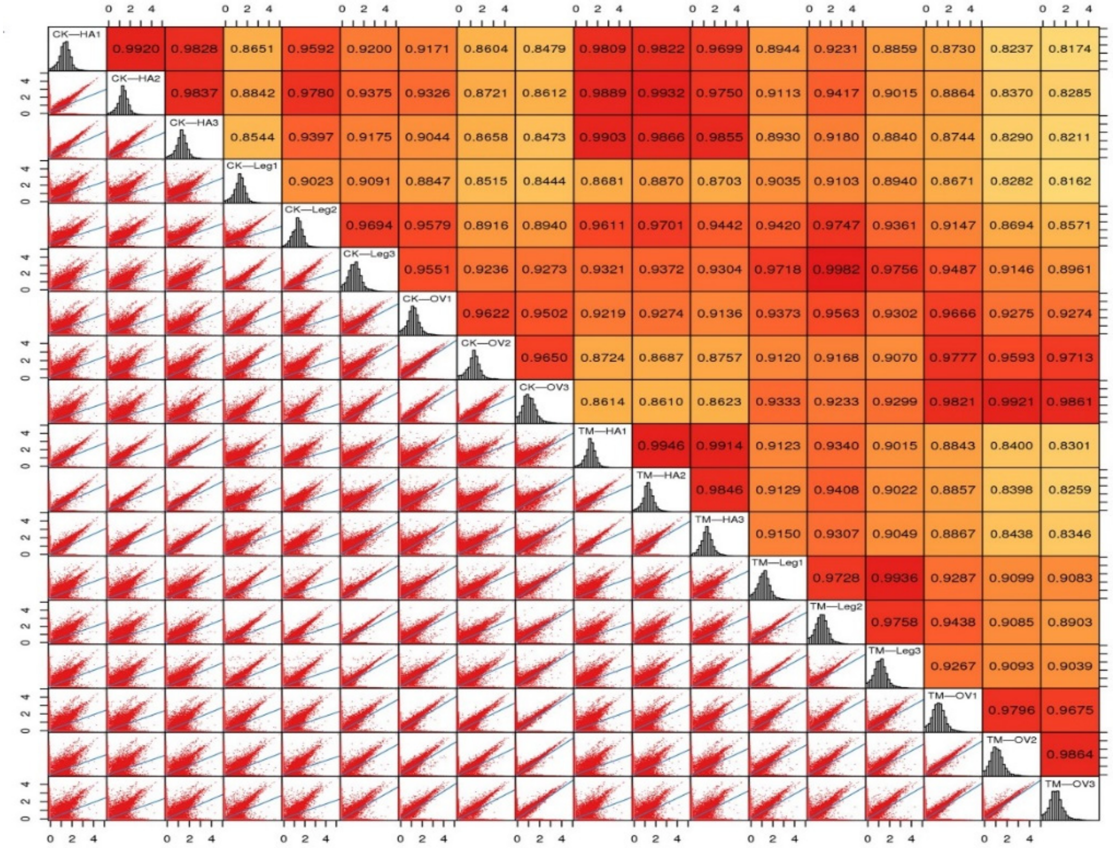

Figure S2. Correlation heat map. Pearson's correlation coefficient (R^2) > 0.8 between biological replicate samples.

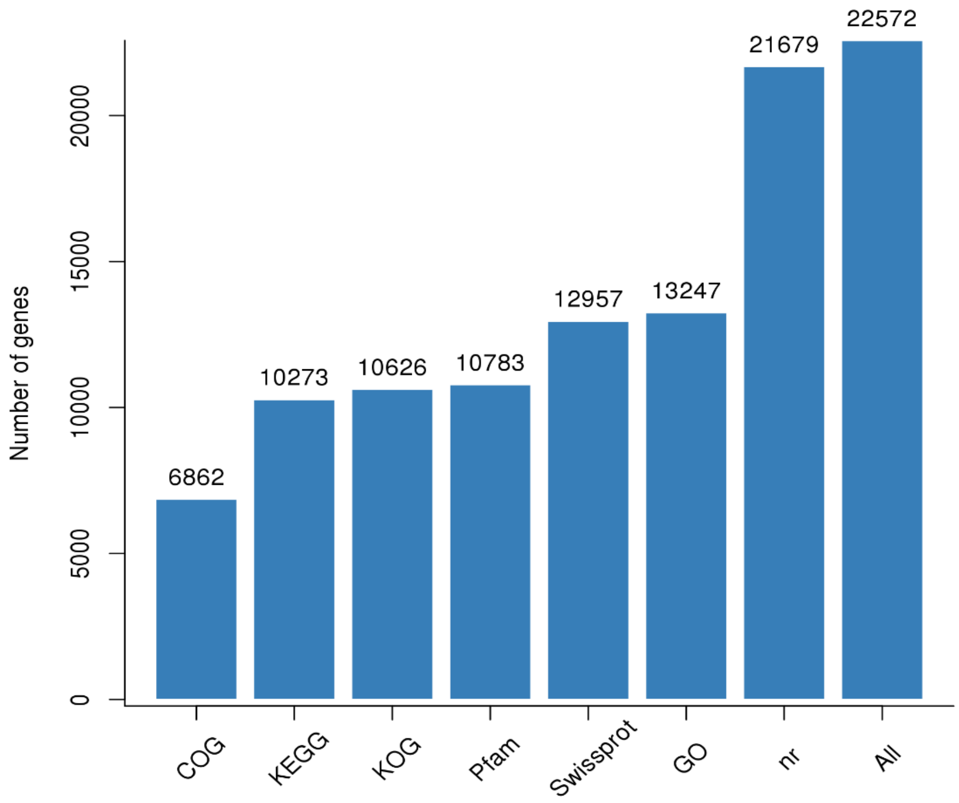

Figure S3. Gene number of function annotation in different databases. These databases include COG, KEGG, KOG, Pfam, Swissprot, GO and Nr.
